# Lateral habenula astroglia modulate the potentiating antidepressant-like effects of bright light stimulation in intractable depression

**DOI:** 10.3389/fphar.2025.1592909

**Published:** 2025-04-23

**Authors:** Sarah Delcourte, Amel Bouloufa, Renaud Rovera, Elie Brunet, Hiep D. Le, April E. Williams, Satchidananda Panda, Rihab Azmani, Olivier Raineteau, Ouria Dkhissi-Benyahya, Nasser Haddjeri

**Affiliations:** ^1^ Univ Lyon, Université Claude Bernard Lyon 1, Inserm U1208, Stem Cell and Brain Research Institute, Bron, France; ^2^ Regulatory Biology Laboratory, Salk Institute for Biological Studies, La Jolla, CA, United States

**Keywords:** refractory depression, bright light stimulation, ketamine, chemogenetic, animal model, rods

## Abstract

**Background:**

Beside image vision, light plays a pivotal role in regulating diverse non-visual functions, including affective behaviors. Recently, bright light stimulation (BLS) was revealed to be beneficial for treating non-seasonal depression, although its mechanism of action is not fully understood.

**Methods:**

We developed a novel mouse model of refractory depression, induced through social isolation and chronic despair during the active (dark) phase of the animal, and we have tested if antidepressant treatments, including BLS, could protect against anxio-depressive-like behavior.

**Results:**

We report that anxiety- and depressive-like behaviors are resistant to BLS as well as to both conventional and new antidepressants, including ketamine. Remarkably, we unveil that BLS potentiates the effect of antidepressants, and this beneficial effect is mediated via rod retinal photoreceptors. Furthermore, we demonstrate that both chemogenetic activation of lateral habenula (LHb) astroglia and serotonin (5-HT) depletion prevent the potentiating effect of BLS on chronic despair.

**Conclusion:**

These results reveal, for the first time, that BLS enhances the efficacy of antidepressants through an unexpectedly circuit involving rods, LHb astroglia and 5-HT.

## Graphical Abstract

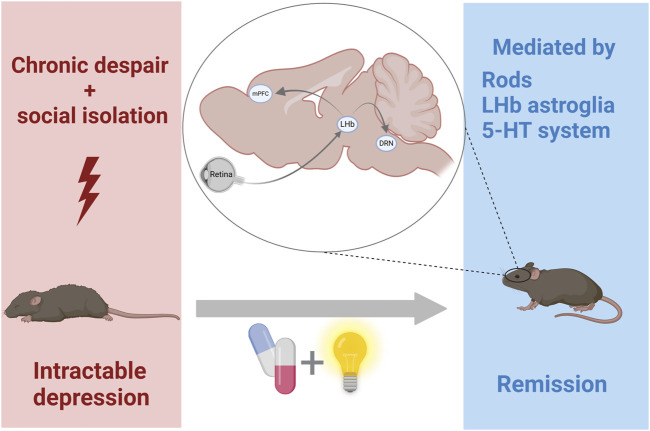

## 1 Introduction

With a lifetime prevalence rate of approximately 15%–20% in the general population, major depression (MD) is the most common psychiatric disorder (Belmaker and Agam, 2008). According to the most recent Global Burden of Disease Study, MD is nowadays the leading cause of disability worldwide (World Health Organization WHO, 2017) and is associated with impaired quality of life, increased risk of mortality, and societal burden (Greenberg et al., 2015). Although depression represents a major economic burden for society, treatments remain unsatisfactory despite a range of pharmacotherapies including the most commonly prescribed class of selective serotonin (5-HT) reuptake inhibitors (SSRI) such as fluoxetine (Prozac). Nonetheless, patients often require several different pharmaceutical agents to achieve a beneficial effect, and only about two-thirds of patients experience a clear favourable response (Papakostas and Ionescu, 2015). In addition to a therapeutic delay of up to several weeks, a significant number of patients express significant residual symptoms that are inadequately treated with current antidepressants (Conradi et al., 2011).

One of the safer, low-cost, non-invasive and underestimated therapeutic candidate is bright light stimulation (BLS). Indeed, light plays a critical role in health, acting directly or through the circadian system to modulate brain structures involved in sleep regulation, mood and cognition (Legates et al., 2014). Light exerts both positive and negative effects depending on the characteristics of the light exposure (timing, duration, intensity, spectrum) (Dkhissi-Benyahya et al., 2000). Hence, aberrant light cycles, through reception of melanopsin retinal ganglion cells, produce depressive-like behaviors and impair cognition in animals and in humans (Legates et al., 2012; Fernandez et al., 2018). Moreover, the prevalence of MD is increased in night-workers, in whom both light exposure and circadian rhythmicity are altered. Inversely, depressed patients frequently display disturbed circadian rhythms and sleep/wake cycles (McClung, 2013). Clinically, the antidepressant efficacy of BLS has been revealed over the past 3 decades in the treatment of seasonal depression (Wirz-Justice et al., 2004). More recently, BLS treatment has also proven to be effective in non-seasonal depression (Menegaz de Almeida et al., 2025) and surprisingly even more efficient than Prozac (Lam et al., 2016).

Despite a large body of evidence demonstrating its beneficial effects, the neurobiological mechanisms through which light acts are not yet fully understood. In recent studies, the lateral habenula (LHb), a part of the epithalamus, has been shown to play a critical role in producing behavioral substrates of susceptibility and in encoding stress signals (Huang et al., 2019; Zhukovskaya et al., 2024). In particular, an excessive bursting activity in the LHb has been associated with depressive-like symptoms (Yang et al., 2018; Lin et al., 2022). Interestingly, decreasing bursting activity in specific LHb neuron subpopulations that project to dorsal raphe serotoninergic (5-HT) neurons is required to reduce despair-like behaviors and underlies the therapeutic effects of BLS on depressive behaviors (Liu et al., 2024). Similarly, the fast-acting antidepressant ketamine rapidly elevates mood by blocking NMDA receptors-dependent bursting activity in LHb neurons (Yang et al., 2018). Conversely, dorsal raphe 5-HT nucleus suppresses the excitability of LHb neurons, supports vicarious emotions and leads to resilience by tuning definite patterns of habenular neuronal activity (Mondoloni et al., 2024). Importantly, stress-induced activation of the LHb involves astroglia in producing depressive-like behaviors (Cui et al., 2018). However, the cellular mechanisms underlying the therapeutic effect of light in resistant depression have not yet been studied.

The main objective of this study was to assess the involvement of the habenular and 5-HT systems, two important interactive brain networks regulating mental health (Huang et al., 2019) that have been suggested to play a key role in mood regulation by light (Legates et al., 2014; Metzger et al., 2017; Fernandez et al., 2018). Notably, most current animal models of depression do not exhibit resistance to many existing treatments. Developing a model of refractory depression is essential for understanding the mechanisms underlying treatment resistance in humans, testing novel therapeutic approaches and drugs, identifying new drug targets, and ultimately improving personalized treatment strategies. Using a novel model of refractory depression, we show for the first time that BLS, through rod photoreceptors, potentiates the effects of a combination of fast-acting antidepressants. Singularly, we uncover a modulatory role for LHb astroglia and a permissive action of 5-HT neurotransmission in the enhancing effect of BLS.

## 2 Methods

### 2.1 Animals

This research was meticulously conducted under the highest ethical standards. All animal procedures were in strict accordance with current national and international regulations on animal care, housing, breeding, and experimentation were approved by the regional ethics committee CELYNE (C2EA42-13-02-0402-005), by the French Ministry of Higher Education, Research and Innovation (APAFIS #41108 & #1128), the European Directive 2010/63/EU and ARRIVE guidelines. All efforts were made to minimize suffering. Six-week-old male C57BL/6J wild-type mice and two photoreceptor-deficient mice were used: *Opn*_*4*_^*−/−*^ knockout for melanopsin (Hattar et al., 2003) and *Nrl* ^*−/−*^ (*Neural retina leucine zipper)* mice characterized by the complete loss of rods (Mears et al., 2001). Mice were individually housed in a temperature- and humidity-controlled environment under a 12 h light/12 dark (12L/12D, 50 lux) cycle. All the behavioural tests were conducted during the active phase of the animals, 2 hours after lights off (zeitgeber time 14, ZT14), under dim red light. Mice received food and water *ad libitum* and were allowed 2 weeks to acclimate before surgery.

### 2.2 *In vivo* chemogenetic experiment

Mice were anesthetized with urethane (1.3 g/kg, i.p.; Sigma) and Xylazine (12 mg/kg i.p.; Bayer), placed in a stereotaxic frame and body temperature was monitored and maintained at 37°C–38°C with an electric heating pad. They were implanted with cannulas for the virus microinjections. Briefly, 0.2 μL of ssAAV-5/2-hGFAP-hM3D (Gq)-mCherry-WPRE-hGHp(A) (GFAP-Gq-DREADD; Viral vector Facility- Zurich University) was infused bilaterally into the LHb (antero-posterior + 4.24 mm; medio-lateral ± 0.48; dorso-ventral + 4.2 mm from bregma with a 45° angle) using 33 gauge injectors at rate of 0.05 μL/min. Control group received the same surgery and were infused with vehicle. The experiments were carried out 10 days’ post-surgeries.

### 2.3 Immunohistochemical staining

Animals received pentobarbital (50 mg/kg, i.p.) and were transcardially perfused with 4% paraformaldehyde (PFA). Brains were removed and post-fixed in 4% PFA at 4°C for an additional 24 h, rinsed in phosphate-buffered saline (PBS, pH 7.4), and cryo-protected in 30% sucrose in PBS 0.1 M for an additional 48 h at 4°C. Brains were sliced at 30 μm thickness and sections were incubated for 24 h with anti-GFAP (mouse, 1:500, Sigma), anti-NeuN (guinea Pig, 1:500, Synaptic systems) and anti-RFP (rabbit, 1:1000, MBL) antibodies in PBS with 0.4% triton (PBST) and 5% normal goat serum. RFP signal was amplified using an anti-rabbit biotinylated secondary antibody (1:200, Vector Biosystem) and streptavidin-DTAF complex (1:250, Jackson Labs Technologies) for 30 min. Glial fibrillary acidic protein (GFAP), neuronal nuclear protein (NeuN), and red fluorescent protein (RFP) stainings were visualized using respectively Alexa-488, Alexa-647 or Streptavidin-Cy3 (1:500, Jackson Immunoresearch). Images were obtained with a Leica SP5 confocal (Leica Microsystems). Z-series images were taken at 2 µm intervals.

### 2.4 Chronic despair model of resistant depression (CDMRD)

At ZT14, mice were placed in a tank filled with water (25°C) and chronic depression-like behavior was induced by subjecting mice to repeated swim sessions 10 min daily for 5 consecutive days (Serchov et al., 2015; Sun et al., 2015; Delcourte et al., 2017b). The Immobility time was analyzed during the first 4 min. Then, every week, and during the 4 weeks post-CDMRD, immobility time was recorded after a swimming passive stress session performed at ZT14. The repeated exposure to swimming at ZT14 leads to a long-lasting increase of the immobility time (Delcourte et al., 2017b). Importantly, both muscarinic and NMDA receptor antagonists (including scopolamine and ketamine) have been used for decades in animals to impair their performance in a variety of tasks requiring intact working and reference memory (Buccafusco, 2008; Farahmandfar et al., 2016). If the passive stress coping (and the chronic despair in presence of BLS) was solely considered as resulting of coping with the forced swim stressor as recently suggested (Molendijk and de Kloet, 2019), one may assume that scopolamine and ketamine, at doses that induce amnesia, should rather decrease active coping behavior (swimming and climbing) and not the opposite. As previously discussed (Molendijk and de Kloet, 2019), the repetitive predictable swim stress used in the CDMRD is designed to mimic everyday human stress, such as daily repetition of a unescapable stressful situation.

### 2.5 Sucrose preference test

Mice were habituated to 2 bottles of 1% sucrose during 24 h. Then, they were given the choice to drink from 2 bottles (1% sucrose solution and tap water bottle) during 72 h. The positions of the bottles in the cage were switched every 24 h to avoid possible side-preference effects.

### 2.6 Elevated plus maze

Animals were placed in the room 30 min before the beginning of the test. Experiments were performed during the dark phase of the 12L/12D cycle, at ZT14. The apparatus consisted of 2 plexiglass open arms (6 × 96.5 cm), and 2 closed arms (6 × 95.5 cm), surrounded by 15 cm black high walls elevated 50 cm above the floor. At the beginning of the test, the mouse was placed in the central platform of the maze, facing an open arm. Each session lasted 6 min and was video recorded to analyze the time spent in the open arms.

### 2.7 Daily locomotor activity recording

As previously assessed (Dkhissi-Benyahya et al., 2007), for monitoring locomotor activity, wild-type mice were housed individually in cages equipped with passive infrared motion captors and a computerized data acquisition system (CAMS, INSERM). Activity records were analyzed using ClockLab software (Actimetrics). The rhythms of locomotor activity were recorded from the beginning to the end of the experiment.

### 2.8 Light stimulation protocols

At the end of the 5 days of CDMRD protocol, mice were exposed every day to a bright warm white stimulation (BLS, 1000 lux; LED bulb) for 1 hour at ZT11 during all the duration of the protocol (as shown insert in figures). The effect of BLS on depressive-like behavior was analyzed using the passive stress coping (PSC) test performed at ZT14, once a week, during the 33 days post-CDMRD. To analyse the potential efficacy of bright white light stimulation on behavioral despair in the mouse, light intensities typically range from 1,000 to 3,000 lux (Kawai et al., 2015; Huang et al., 2019; Wang et al., 2023). Since nocturnal rodent have an innate aversion to bright light, we chose the lowest intensity level to assess its potential efficacity in treating depressive-like behavior. Mice were housed individually in plexiglass cages with white LED bulbs placed 7 inches above the cages. The intensity level was measured at the bottom of the cage, at the mouse level, using a luxmeter.

### 2.9 Drug treatment

Ketamine hydrochloride and escitalopram were purchased from Sigma-Aldrich. Scopolamine hydrobromide and Clozapine-N-oxide (CNO) were purchased from Tocris Biosciences. All drugs were dissolved in a 0.9% saline solution. Escitalopram was administered at 10 mg/kg, i.p during 5 consecutive days following the CDMRD, or during the 5 days preceding the passive stress coping for the naïve group. Ketamine hydrochloride was administered acutely at 3 mg/kg (i.p.) in combination with scopolamine (0.1 mg/kg, i.p), 30 min before the passive stress coping test at day 33. CNO was dissolved in saline and administered at 1 mg/kg (i.p.) 30 min before the passive stress coping test (Delcourte et al., 2023).

Before the 33-day swimming test, animals were injected 3 days with 150 mg/kg of 4-Chloro-DL-phenylalanine (PCPA, Sigma Aldrich), the last day of injection was 24 h before the last passive stess coping test as previously described (Etiévant et al., 2015).

### 2.10 Plasma corticosterone radioimmunoassay

Two groups of singly housed wild-type mice were euthanized by cervical dislocation, the first one the day before performing the CDMRD test and the second group the day after the last swimming session. These 2 groups were compared to non-isolated naïve mice. Blood sampling was performed between ZT9-ZT11 during the light phase and corticosterone was quantified using an ELISA kit (Arbor Assays) according to the manufacturer’s instructions.

### 2.11 Statistical analysis

All statistical analyses were performed with Statistica Software. For data normally distributed, One-way ANOVA followed by *post hoc* LSD Fischer test was performed for comparison between groups. To compare immobility for the same animal across days, one-way repeated measures ANOVA with Wilcoxon *post hoc* test was performed. Comparisons of the mean between 2 groups were analyzed via Student’s t tests. Values are presented as mean ± standard error of mean (S.E.M), with consideration of p < 0.05 as the threshold of statistical significance.

## 3 Results

### 3.1 The CDMRD recapitulates behavioral hallmarks of refractory depression

**FIGURE 1:**
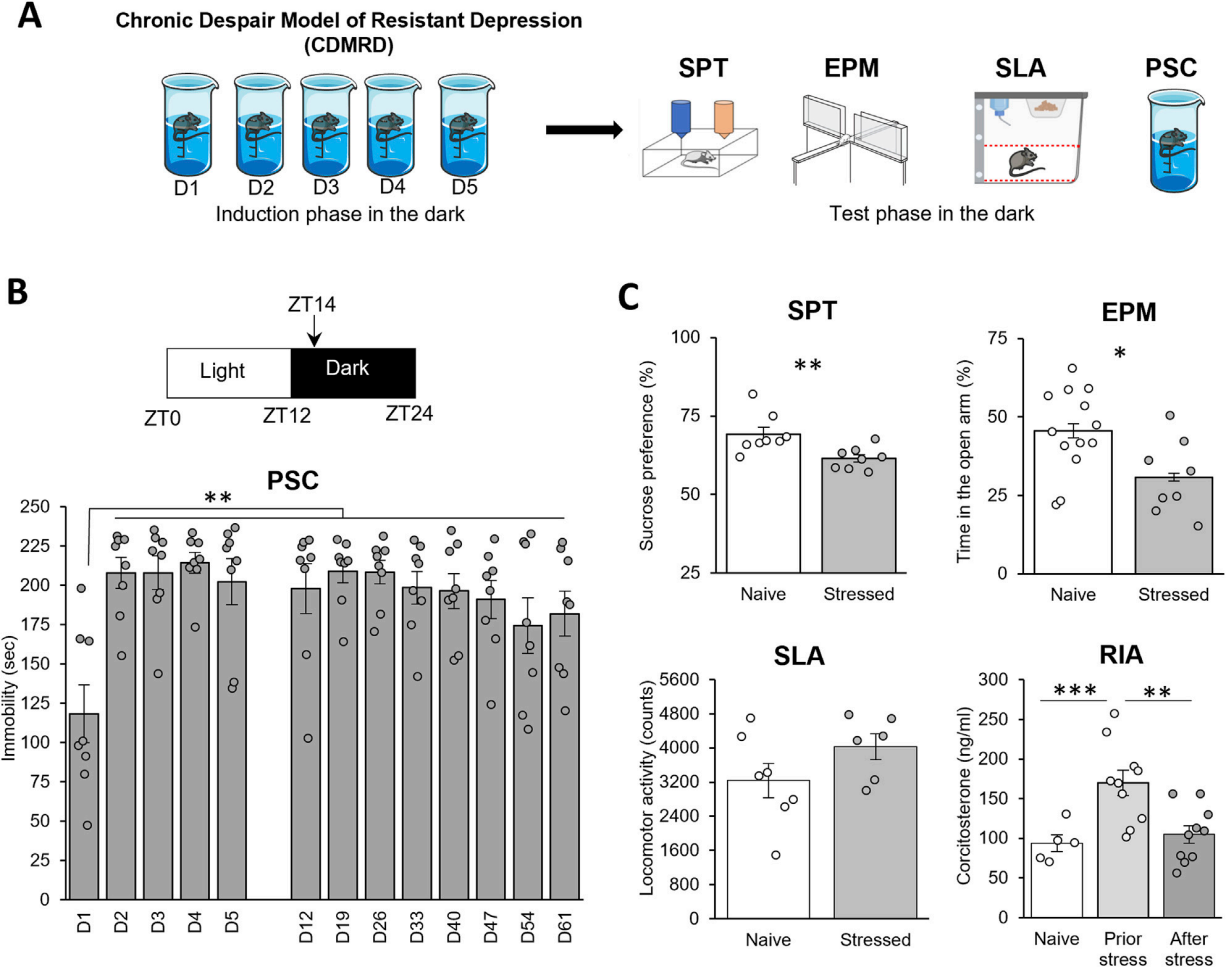
CDMRD: a pharmaco-resistant model of depression: **(A)** Schematic overview of the experimental design: CDMRD paradigm, followed by the test phase. **(B)** CDMRD: 6-weeks old C57BL/6J male mice are exposed to a 12L/12D cycle, with light on (50 lux) at zeitgeber 0 (ZT0) and off at ZT12. Animals were forced to swim on 5 successive days [D] for 10 min at ZT14 and a passive stress coping (PSC) was performed every week during 8 weeks (n = 8) at ZT14. One-way repeated measures ANOVA with *post hoc* Wilcoxon Test, **p < 0.01 vs. D1. **(C)** Sucrose Preference Test (SPT): Naïve and stressed mice (48 h post-stress) were subjected to the sucrose preference test (n = 8). Elevated plus maze (EPM): naïve and stressed mice (1-week post-stress) were subjected to the elevated plus maze (n = 8–13). Spontaneous locomotor activity (SLA) of mice 24 h before the last PSC test (n = 6–7). Plasma corticosterone profile measure by radioimmunoassay (RIA) in naïve grouped mice (n = 5), prior stress (n = 10) or after CDMRD (n = 10). LSD test: *p ≤ 0.05; **p ≤ 0.01; ***p ≤ 0.001.

We first refined a previously reported validated model of depression (Warden et al., 2012; Serchov et al., 2015; Sun et al., 2015; Bullich et al., 2021; Moliner et al., 2023; Sarrazin et al., 2024; Sekssaoui et al., 2024). Hence, 6-week-old C57BL/6J male mice, socially isolated, were forced to swim during their active phase, at zeitgeber time 14 (ZT14, 2 h after light OFF) on 5 successive days for 10 min (Figure 1A) and were then tested once a week. Therefore, such a stress performed at ZT14 was extremely effective to elicit a long-lasting (up to 8 weeks) depressive-like behavior (Figure 1B; *p ≤ 0.01*), in agreement with previous studies showing that repeated daily stress exposure has a more negative outcome when applied during the dark/active phase (Bartlang et al., 2012; Serchov et al., 2015; Sun et al., 2015; Delcourte et al., 2017a). Interestingly, CDMRD mice expressed a reduced sucrose preference when compared to naive mice (*p ≤ 0.01*), indicating that, in addition to behavioral despair, CDMRD also caused anhedonia (Figure 1B). Moreover, the stressed mice exhibited higher anxiety levels, measured as reduced time spent in the open arms in the elevated plus maze (*p ≤ 0.05*). Notably, CDMRD mice displayed lower plasma corticosterone levels, by comparison to isolated-housed control mice (*p ≤ 0.01*), whereas their spontaneous locomotor activity was unchanged.

### 3.2 The CDMRD is resistant to antidepressant’s treatment

**FIGURE 2:**
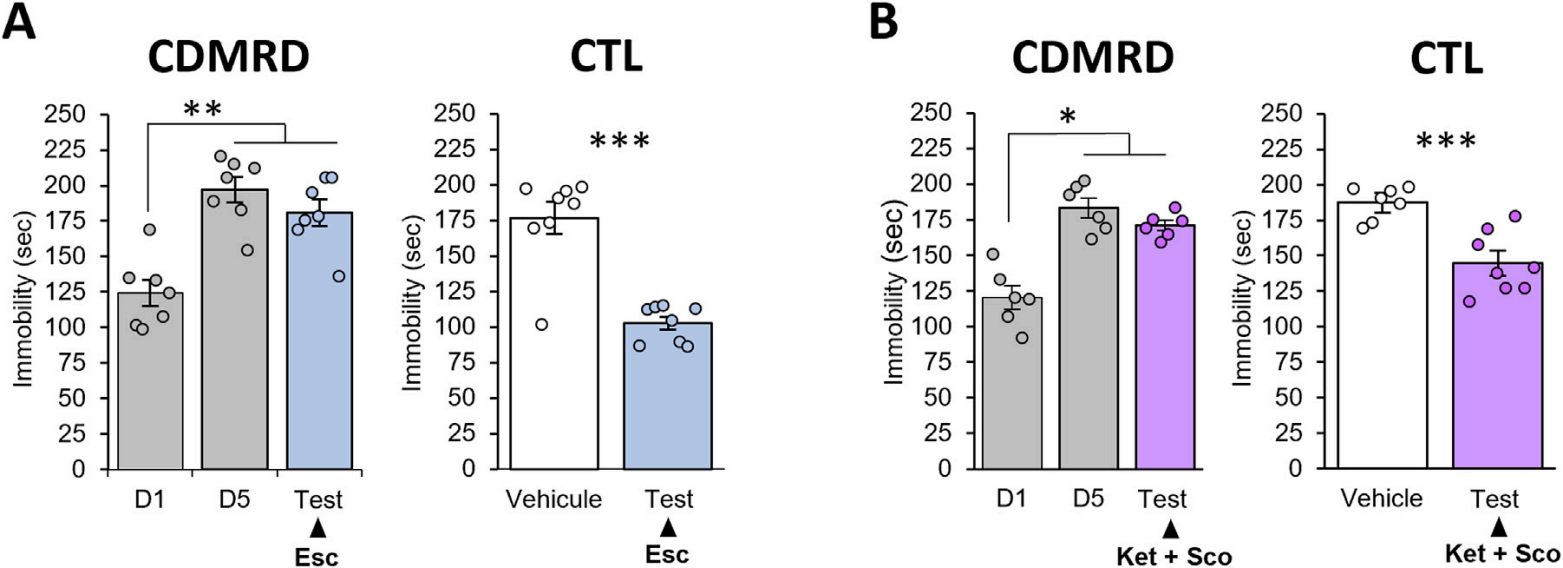
Lack of antidepressant action in CDMRD mice. **(A)** Following CDMRD, mice exposed to a 12L/12D cycle (50 lux during the light phase) were treated with the SSRI escitalopram (10 mg/kg i.p), 5 consecutive days from D10 to D15. A passive stress coping (PSC) test was then realized at ZT14, one-way ANOVA with *post hoc* LSD Test, **p < 0.01 vs. D1. Antidepressant-like effect of a sub-chronic treatment of escitalopram in non-stressed mice (CTL): naïve mice were treated with vehicle or the SSRI escitalopram (10 mg/kg) during 5 consecutive days. On day 5, they received the last injection 30 min before the PSC test. (n = 8) unpaired Student’s t-tests; ***p < 0.001. **(B)** Following CDMRD, mice exposed to a 12L/12D cycle (50 lux during the light phase), received an acute injection of ketamine (Ket, 3 mg/kg, i.p.) and the muscarinic antagonist scopolamine (Sco, 0.1 mg/kg, i.p) 4 weeks post-stress, at day 33 (D33). Antidepressant-like effect of a combination of Ketamine + Scopolamine in non-stressed mice (CTL): naïve mice were submitted to a PSC test. 24 h later, the mice were injected with vehicle or a combination of ketamine (3 mg/kg, i.p) and the muscarinic receptor antagonist scopolamine (0.1 mg/kg, i.p), 30 min before the second PSC test (n = 7–8), *p < 0.05 and ***p < 0.001 using unpaired Student’s t-tests. Data are expressed as means ± S.E.M.

Using passive stress coping test as a main antidepressant readout, we found that treatments with several classes of antidepressants including the selective 5-HT reuptake inhibitor (SSRI) antidepressant escitalopram (Figure 2A), the combination of amnesiac-combo of the NMDA receptor antagonist ketamine and the muscarinic antagonist scopolamine (Figure 2B), ketamine alone (Supplementary Figure S1) or of the NMDA receptor modulator GLYX-13 (Supplementary Figure S2) failed to reverse the behavioral despair in CDMRD stressed mice (*p ≥ 0.05*). However, escitalopram or the combined treatment of ketamine and scopolamine or ketamine alone or GLYX-13 were effective in naïve mice (Figure 2; Supplementary Figures S1, S2), thus revealing CDMRD as a robust pharmaco-resistant model.

### 3.3 BLS potentiates the antidepressant response in CDMRD

**FIGURE 3:**
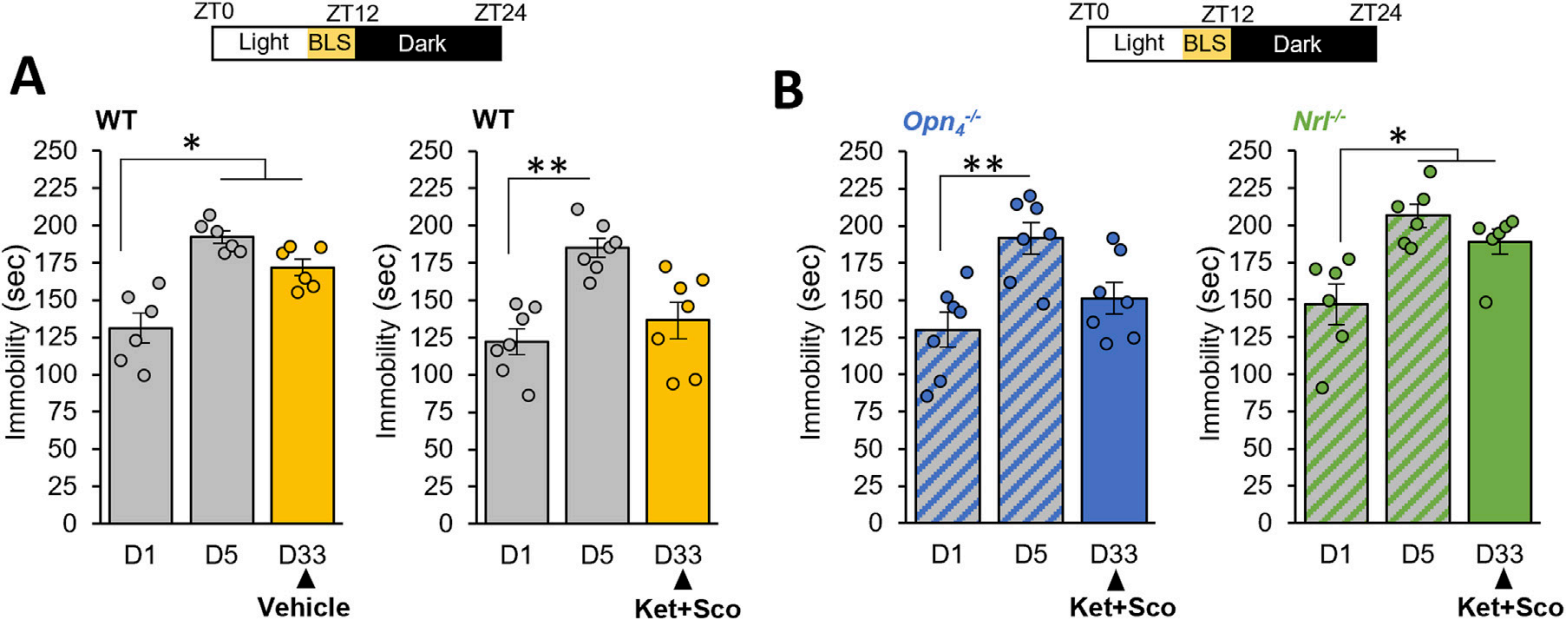
BLS, through rods, potentiates the antidepressant response of a combination of ketamine and scopolamine: **(A)** The day after the last day of CDMRD induction protocol in wild-type (WT) animals, a bright white light stimulation (BLS, 1000 lux), 1 h per day from ZT11 to ZT12 (light on from ZT0 to ZT12) was administered to the CDMRD mice **(A)**, (n = 6). One-Way repeated measures ANOVA with *post hoc* Wilcoxon test. *p < 0.05 vs. D1. CDMRD wild-type (WT) animals **(A)**, (n = 7), melanopsin knockout (*Opn4* ^*−/−*^, **(B)**, n = 7) and rod-deficient mice (c, *Nrl* ^*−/−*^, **(B)**, (n = 6) housed under a 12L/12D cycle, are exposed to an additional 1-h BLS from ZT11 to ZT12 every day until the end of the protocol. 4 weeks post-stress, on day 33 (D33), all genotypes received an acute injection of Ketamine (Ket, 3 mg/kg, i.p) and Scopolamine (Sco, 0.1 mg/kg, i.p). Note that the augmenting effect of BLS was absent in *Nrl* ^*−/−*^ mice. One-Way repeated measures ANOVA with *post hoc* Wilcoxon test. *p < 0.05; **p < 0.01 vs. D1.

We then explore the beneficial effects of BLS on this model. Mice were exposed daily for 1 hour during the light phase (before light off) to white warm BLS (1000 lux) up to day 33; and similarly, BLS failed to produce an antidepressant response (Figure 3A; *p ≥ 0.05*) and to alter spontaneous locomotor activity (Supplementary Figure S3). Importantly, we found that a 4-week exposure to BLS, in combination with acute ketamine and scopolamine administration, reduced the immobility time observed in the passive stress coping test, demonstrating unequivocally that BLS potentiated the antidepressant response of the pharmacological treatment (Figure 3A; *p ≤ 0.05*).

While the source of photic information affecting mood involves intrinsically photosensitive retinal ganglion cells (ipRGCs) expressing melanopsin (Legates et al., 2012; Fernandez et al., 2018), recent studies reveal that rods can drive non-image forming responses to light, in particular under high light intensity (Calligaro et al., 2019; Beier et al., 2022). To determine the retinal photoreceptor implicated in BLS potentiating effect on the behavioral phenotype, we use melanopsin (*Opn4* ^*−/−*^) and rod (*Nrl* ^*−/−*^ *)* photoreceptor-deficient mice. Unexpectedly, we showed that *Opn4* ^*−/−*^mice that underwent CDMRD protocol retained the potentiating action of BLS (Figure 3B) whereas rodless mice remarkably lost this effect (Figure 3B; *p ≤ 0.01*).

### 3.4 The augmentation of the antidepressant response induced by BLS is modulated by astroglia LHb and 5-HT neurotransmission

**FIGURE 4:**
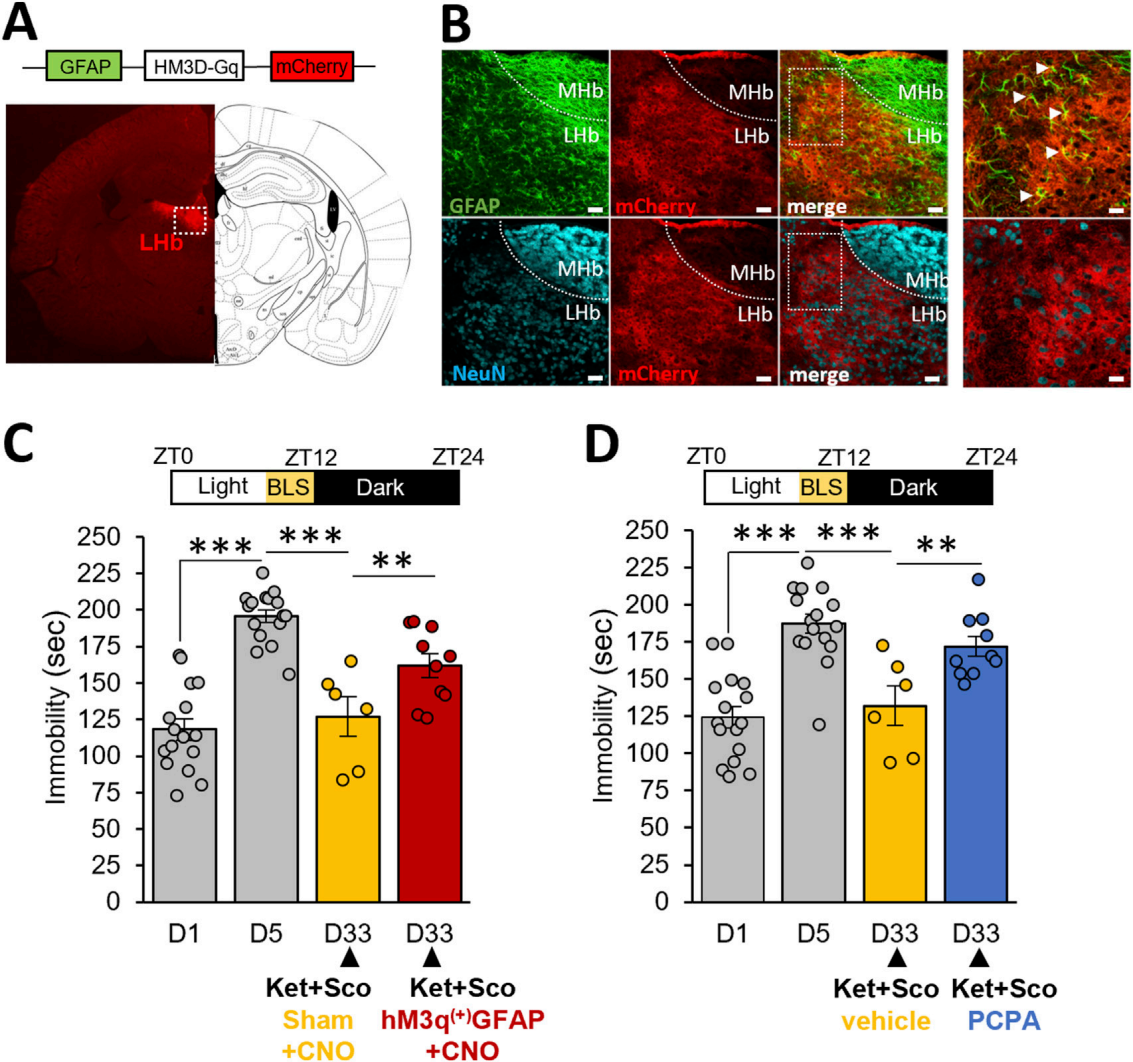
Role of LHb astrocytes and 5-HT system in the antidepressant effect of BLS: **(A)** Following CDMRD, mice were intracerebrally injected with vehicle or a AAV hM3q^(+)^-GFAP-Gq-mCherry virus in the LHb. Coronal image and scheme of brain section showing m-Cherry labelling (red) in the LHb (white square). **(B)** Astroglial and neuronal staining in the LHb: Anti-GFAP (green) and anti-NeuN (blue) immunohistochemistry confirming expression of AAV hM3q^(+)^-GFAP-Gq-mCherry virus in astroglia but not in neurons. scale bar = 150µm, enlargement, scale bar = 75 µm. **(C)** CDMRD mice were intracerebrally injected with vehicle or a AAV hM3q^(+)^-GFAP-Gq-mCherry virus in the LHb. A 4-week BLS was then applied. At day 33 (D33), mice received an injection of Clozapine-n-oxide (CNO, 1 mg/kg, i.p.) 1 h before the passive stress coping test. Half an hour after the CNO injection, the combination of ketamine/scopolamine (Ket + Sco, respectively 3 and 0.1 mg/kg, i.p) was administered to mice (n = 6–16). ***p ≤ 0.001 vs. D1. On D33, the two groups (sham and AAV-injected) were compared using LSD tests. **p ≤ 0,01. **(D)** CDMRD mice were subjected to the same BLS paradigm previously described. Three days before the passive stress coping test at D33 (ZT14), mice (n = 16) were divided into 2 groups, one received a dose of 150 mg/kg/day (i.p) of para-chlorophenylalanine (PCPA) to reduce the levels of 5-HT whereas the control group received a 0.9% saline injection (n = 6–10). At D33, both groups received the combination of Ket + Sco (respectively 3 and 0.1 mg/kg, i.p). **p ≤ 0.01; ***p ≤ 0.001 vs D1. On day 33, the two groups (vehicle and PCPA) were compared using LSD tests.

Given the crucial involvement of LHb glia in depressive-like behavior, we sought to determine whether selective chemogenetic activation of transduced astroglia in LHb, counteracted the potentiating antidepressant action of BLS in CDMRD mice. Injection of the GFAP-Gq-DREADD virus in the LHb (Figure 4A) showed fields of mCherry-positive cells, that consistently express the GFAP astroglia marker and lack the NeuN neuronal marker (Figure 4B). Remarkably, Clozapine-n-oxide (CNO) administration, by producing a selective and robust activation of expressing Gq-DREADD LHb astrocytes (de Ceglia et al., 2023), significantly prevented the potentiating effect of BLS on the action of the ketamine/scopolamine combination (Figure 4C, *p ≤ 0.01*). Interestingly, several lines of evidence significantly link the effects of light to 5-HT and LHb regulation (Liu et al., 2024). Accordingly, we demonstrated that PCPA pre-treatment prevented the potentiating effect of BLS on the action of the ketamine/scopolamine combination, pointing out a permissive role of the 5-HT system in the antidepressant behavioral phenotype in this CDMRD mouse (Figure 4D; *p ≤ 0.01*).

## 4 Discussion

The current results underscore the importance of the system/circuit involving rods, LHb astroglia and 5-HT in the development of CDMRD-induced depressive-like symptoms and the therapeutic potential of BLS. To date, CDMRD is one of the few reliable mouse models for refractory depression, primarily based on chronic despair during the active (dark) phase of the animal and combined to social isolation. As reported in our previous study (Bullich et al., 2021), we propose here that the timing of stress, testing and animal housing are critical factors for the CDMRD in mice. Accordingly, we found that adult male mice required social isolation and forced swimming during their active phase for five consecutive days, followed by weekly testing. Therefore, such a potent stress at ZT14 effectively induced a long-lasting depressive-like phenotype (up to 2 months), characterized by behavioral despair, anhedonia and higher anxiety levels. Notably, CDMRD mice displayed lower plasma corticosterone levels, suggesting blunted HPA-axis reactivity while their spontaneous locomotor activity remained unchanged, ruling out potential false positives in the passive stress coping test (Sotnikov et al., 2014). Using this stress coping test as a main antidepressant readout, we next found that treatments with several classes of antidepressants failed to induce an antidepressant-like response. Likewise, treatment with escitalopram or with ketamine, whether given alone or in the combination of amnesiac-combo of ketamine and scopolamine, or with the NMDA receptor modulator GLYX-13 failed to reverse the behavioral despair in stressed mice. Hence, CDMRD represents a novel treatment-resistant depression-like animal model exhibiting construct (corticosterone dysfunction), face (change in despair-, anhedonia- or anxious-like behavior) and predictive (reversal only with combinatory treatment) validity (Planchez et al., 2019).

The clinical translational potential of light therapy is vast, offering a non-invasive, affordable, and personalized treatment option for patients with depression and other mental health disorders. Its ability to improve mood, regulate sleep, and modulate brain activity presents a promising avenue to enhance current therapeutic approaches, particularly for those who struggle with treatment-resistant depression. With ongoing research into its mechanisms and effectiveness, light therapy has the potential to become a mainstream treatment for depression and other related conditions, benefiting a wide range of patients across various clinical settings. In addition, the duration and intensity of light exposure for depression research are crucial, as both parameters can significantly influence the therapeutic effectiveness and underlying neurobiological mechanisms. BLS, a form of photobiomodulation, modulates brain activity, and has gained interest in its potential to enhance neuronal activity and mood regulation. However, precise dose response curves for duration and intensity still need to be established to optimize neurotransmitter systems regulation while minimizing potential side effects (overstimulation or tolerance, toxicity). These parameters may need to be adjusted based on individual patient characteristics and specific brain regions targeted during therapy. To explore the beneficial effects of BLS on this model, mice were exposed daily for 1 hour (before light off) to white warm BLS (1000 lux, Kawai et al., 2015); and similarly, BLS given alone neither induced an antidepressant response nor alter spontaneous locomotor activity. Conversely, we found that a 4-week exposure to BLS, in combination with acute ketamine and scopolamine administration, reduced the immobility time observed in the passive stress coping test, demonstrating that BLS unequivocally potentiated the antidepressant response of the pharmacological treatment.

In mammals, light detection occurs exclusively in the retina through three types of photoreceptors, the classical rods and cones and the intrinsically photosensitive melanopsin retinal ganglion cells or ipRGCs. ipRGCs exhibit widespread projections throughout the brain, including regions controlling mood, such as the LHb (Hattar et al., 2006) recently proposed as a key structure in refractory depression (Korlatowicz et al., 2023; Chen et al., 2024). Unexpectedly, we found that melanopsin knockout (*Opn4* ^*−/−*^) mice that underwent CDMRD protocol retained the potentiating action of BLS, while rod photoreceptor-deficient (*Nrl* ^*−/−*^) mice remarkably lost this effect. This result suggests that the potentiating effect of light is not driven by melanopsin intrinsic photosensitivity but required ganglion cells expressing melanopsin. Indeed, ipRGCs integrate input from rods and cones and serve as the final and unique retinal relay for light transmission to multiple brain regions, including the LHb, either directly or indirectly. Interestingly, an indirect pathway from a subset of ipRGCs to GABAergic neurons in the ventral lateral geniculate nucleus and intergeniculate leaflet has been reported, which in turn inhibit the neural activity of LHb (Huang et al., 2019). Accordingly, chemogenetic activation of ipRGCs did not induce FOS expression in the LHb (Milosavljevic et al., 2016). However, given that light-induced mood regulation have been shown to be absent in mice lacking ipRGCs, ipRGCs appear necessary to relay the photic information to the LHb (Güler et al., 2008; Legates et al., 2014; Milosavljevic et al., 2016). The involvement of rods at the relatively high BLS intensity used here aligns with the idea that these cells have the capacity to respond at this level (Lall et al., 2010; Calligaro et al., 2019). While the precise mechanisms by which rod photoreceptors regulate astrocyte activation in the LHb remain to be fully elucidated, and based on our findings, we can only infer that through these connections, rods may modulate LHb activity and astrocyte function in response to BLS. Further experiments will be necessary to dissect the underlying mechanisms.

LHb has emerged as a key structure at the interface between light effects and 5-HT regulation. LHb, which receives projections from the prefrontal cortex (PFC) as well as the ipRGCs, is anatomically and functionally connected to the raphe 5-HT nuclei (Hattar et al., 2006). As recently unveiled (Mondoloni et al., 2024), LHb-raphe nucleus function was recognized to play a role in the pathogenesis of depression. Hence, Yang and colleagues (Yang et al., 2018) elegantly reported that depression-like symptoms are caused by rapid bursts of firing from LHb neurons due to coordinated activity of NMDA receptors and T-type voltage-sensitive calcium channels. Furthermore, Cui et al. (Cui et al., 2018) identified the involvement of LHb astroglia and demonstrated that surrounding astrocytes which express K+ channels containing the protein Kir4.1, rapidly cleared away K+. This facilitates rapid K+ efflux from the neuron and its entry into a state of hyperpolarization, which increases the likelihood of burst firing (Yang et al., 2018). Congruently, photostimulation of astrocytes expressing ChR2 released potassium ions into the extracellular space, which in turn excited the neurons with an increased firing rate in LHb. Moreover, photostimulation of habenular astrocytes exacerbated depression-like phenotypes with exaggerated despair behavior and anhedonia in tail suspension and sucrose preference tests, respectively (Aizawa et al., 2024). Given this crucial involvement of LHb glia in depressive-like behavior, we found that injection of the GFAP-Gq-DREADD virus in the LHb showed fields of mCherry-positive cells that consistently express the GFAP astroglia marker. Remarkably, CNO administration, by producing a selective and robust activation of expressing Gq-DREADD LHb astrocytes (de Ceglia et al., 2023), significantly prevented the potentiating effect of BLS on the action of the ketamine/scopolamine combination. This result reveals a crucial involvement of LHb astroglia in the beneficial action of BLS. Collectively, these results may assume that the regulation of both extracellular glutamate and K+ within the LHb (Cui et al., 2018; Yang et al., 2018) play a key role in the reinforcing effect of BLS in the CDMRD.

Interestingly, several lines of evidence significantly link the effects of light to 5-HT and LHb regulation (Metzger et al., 2017). Notably, retino-raphe signals were shown to modulate dorsal raphe nucleus 5-HT tone and affective behavior (Ren et al., 2013). Therefore, the antidepressant-like effect of several drugs, including ketamine, was abolished by 5-HT depletion produced by the tryptophan hydroxylase inhibitor, PCPA (Gigliucci et al., 2013). The specific effect of PCPA on immobility time in PSC test is still unclear (Gigliucci et al., 2013; Pham et al., 2017). In our study, it may be due to a ceiling effect masking PCPA action. One could infer that any immobility observed following PCPA treatment in the PSC test likely reflects a lack of behavioral flexibility or an impaired coping mechanism. Since 5-HT is known to facilitate behaviors such as exploration, locomotor activity, and problem-solving, its depletion may impair the animal’s ability to engage in active coping or escape behaviors in response to stressors like those presented in the PSC test. Hence, PCPA effect might represent a counteracting influence rather than a direct contribution. Recently, it has been demonstrated that ketamine injection in depressive-like mice specifically blocks NMDARs in LHb neurons whereas conditional knockout of NMDARs in the LHb occluded ketamine’s antidepressant effects and blocked the systemic ketamine-induced elevation of 5-HT in the hippocampus (Chen et al., 2024). In agreement, we demonstrated that PCPA pre-treatment prevented the potentiating effect of BLS on the action of the ketamine/scopolamine combination, pointing out a permissive role of the 5-HT system in the antidepressant behavioral phenotype.

Because the 5-HT system is deeply interconnected with PFC, RNA-seq analysis was performed in this region, one that plays a key role in major depression and antidepressant response, including treatments like ketamine and light therapy (Warden et al., 2012; Lazzerini Ospri et al., 2024). The analysis identified over 350 differentially expressed genes in the PFC following the CDMRD protocol. Functional enrichment analysis revealed significant perturbations in genes related to myelination (Supplementary Figure S4). Further investigation confirmed the enrichment of 33 unique gene transcripts, primarily associated with late-stage oligodendrogenesis (myelination, 59%), rather than early-stage oligodendrogenesis—consistent with findings in the PFC of major depression (MD) patients (Pantazatos et al., 2017) and depression models (Ma et al., 2016). These results further support CDMRD as a robust model of depression. Notably, over 90% of oligodendroglial transcripts perturbed by CDMRD were reversed by co-treatment with ketamine, scopolamine, and BLS, demonstrating for the first time that BLS enhances the effects of pharmacological treatments by modulating the expression of oligodendrocyte-related genes in the PFC.

## 5 Conclusion

The present study highlights for the first time a modulatory control of astroglia LHb as well as a permissive role of 5-HT neurotransmission in the augmentation of the antidepressant response induced by BLS. As a translational outcome, the current study will certainly improve our understanding of the mechanisms involved in light effects on depression to ultimately optimize therapeutic strategies in drug-resistant or refractory depressed patients, including partial responders to ketamine.

## Data Availability

The datasets presented in this study can be found in online repositories. The names of the repository/repositories and accession number(s) can be found below: https://www.ncbi.nlm.nih.gov/geo/query/acc.cgi?acc=GSE143820.
