# Supplementary material for: Lateral habenula astroglia modulate the potentiating antidepressant-like effects of bright light stimulation in intractable depression
Source: Front Pharmacol. 2025 Apr 23;16:1592909. doi: 10.3389/fphar.2025.1592909 (PMC12055791; doi:10.3389/fphar.2025.1592909)
Supplement: Supplementary file 1 [file DataSheet1.docx]

**Supplementary Information**

Figure S1:

Effects of acute ketamine treatment:

CDMRD: 4 weeks following the stress paradigm, mice were treated with the NMDA receptor antagonist ketamine at a dose of 10 mg/kg (i.p, n=6), one-way ANOVA with post-hoc Dunnett’s Test, **p<0.01 vs D1. CTL: Antidepressant-like effect of a ketamine in non-stressed mice: naïve mice were submitted to a PSC test and to another one 24 h later. Mice were injected with vehicle or the NMDA receptor antagonist ketamine (10 mg/kg, i.p) 24 h before the second PSC test (n=13-15) unpaired Student’s t-tests; ** p<0.01.

Figure S2

**CDMRD CTL**


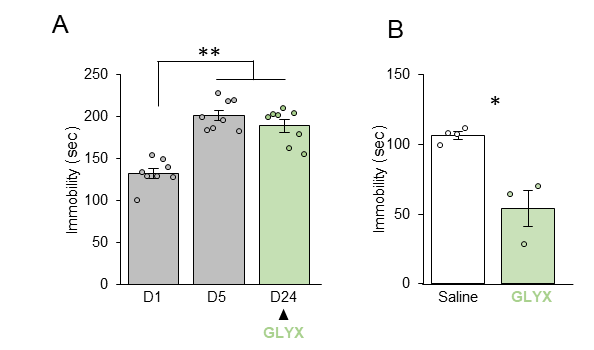


Effects of acute GLYX-13 treatment:

A: Following CDMRD, mice received an acute injection of the NMDA receptor modulator GLYX-13 [GLYX] (10 mg/kg, i.p) and a PSC test was performed 1h later on D24, one-way ANOVA with post-hoc LSD Test, **p<0.01 vs D1 (n=8). B: Antidepressant-like effect of an acute treatment of GLYX-13 in non-stressed mice: naïve mice were treated with vehicle or GLYX-13 [GLYX] (10 mg/kg, i.p). They received the injection 60 min before the PSC test. * p<0.05 using unpaired Student’s t-test (n=3-4).

Figure S3:

ns

Effect of a bright light stimulation on locomotor activity:

After the repeated stress, mice were exposed to an additional BLS (white light, 1000 lux),1 hour per day from ZT11 to ZT12 during 4 weeks or submitted to the classical 12L/12D. The locomotor activity was recorded. ns = non-significant using unpaired Student’s t-test (n=5-6). Data are expressed as means ± S.E.M.

Figure S4:


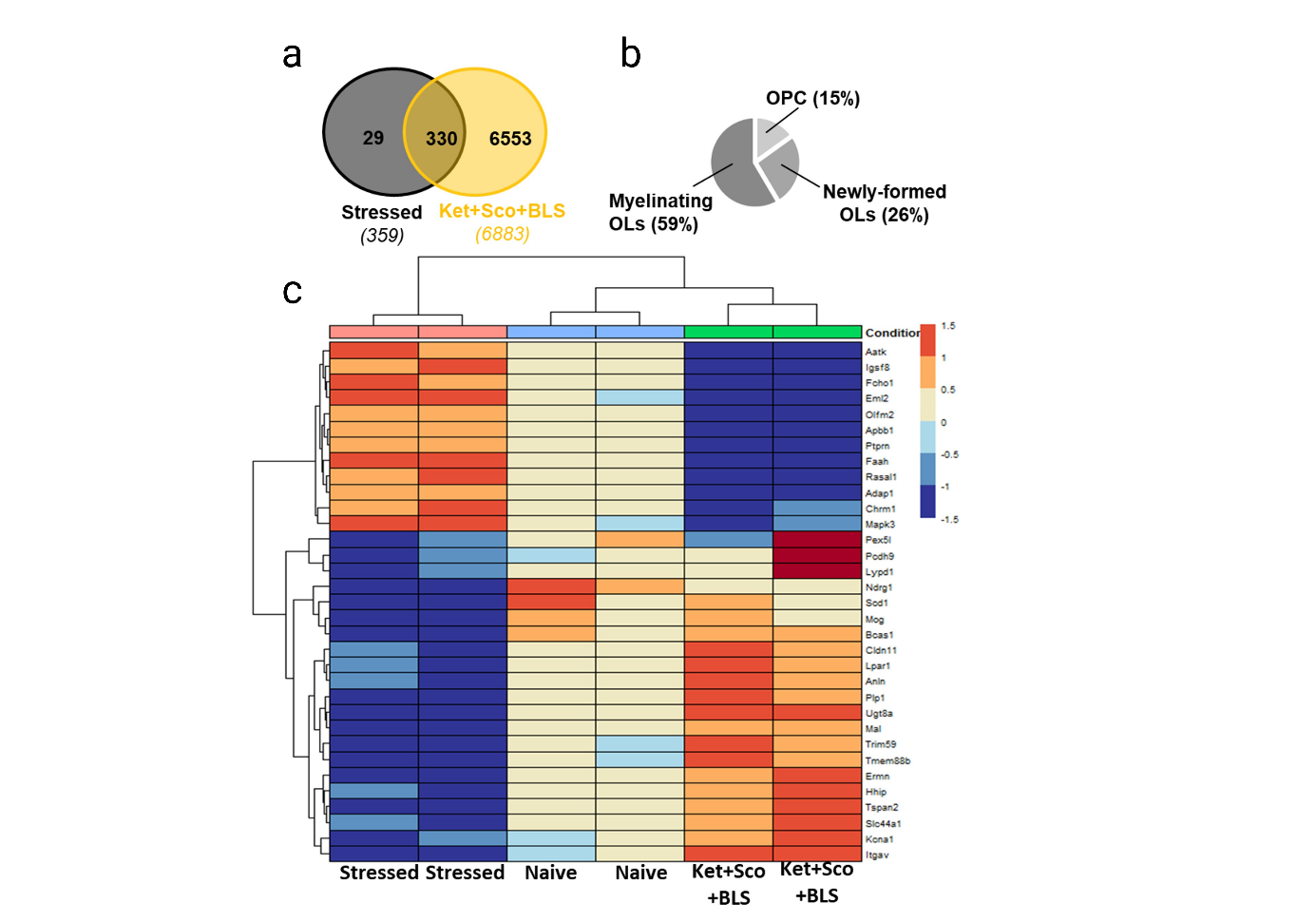


Effect of 5d-RFSS and pharmacological and BLS treatment on gene expression in the PFC

**A**: Venn Diagram illustrating that more than 350 genes were differentially expressed in the prefrontal cortex (PFC) following CDMRD and most (~90%) transcripts altered by CDMRD are modulated by ketamine (Ket), scopolamine (Sco) and BLS co-treatment. **B**: Out of 33 oligodendrocytes (OL) enriched transcripts perturbed by CDMRD, the majority were related to myelinating oligodendrocytes. OPC, OL precursors. (C) Hierarchical clustering of 33 OL-related genes in naïve, stressed and treated animals. Note that treatment (Ket+Sco+BLS) reverts transcriptional oligodendrocytes changes induced by stress.

Supplementary Methods

RNA seq analysis

Two days after the last FST (day 35), mice were euthanized. Both sides of the medial prefrontal cortices were dissected on ice and stored at -80°C. Total RNAs were extracted using Trizol reagent (Invitrogen) as previously described (Calligaro et al., 2019).

*Libraries preparation and high-throughput sequencing*

Libraries were prepared using Illumina’s TruSeq Stranded mRNA HT kit according to manufacturer’s instructions.  In brief, total RNA starting with 700ng was poly-A selected, fragmented by metal-ion hydrolysis and then converted to cDNA using SuperScript II.  Due to the limiting nature of the source tissue, 3 samples had less than 700ng of total RNA for library production. The cDNA was then end-repaired, adenylated and ligated with Illumina sequencing adapters.  Finally, the libraries were enriched by 15 cycles of PCR amplification.  Libraries were pooled and sequenced using an Illumina HiSeq 2500 with 50-bp single-read chemistry.

*Transcriptome data analysis*

Whole-transcriptome profiling of prefrontal cortices was performed by RNA-Seq. Sequenced reads were mapped to the reference mouse genome using (org.Mm.eg.db, <http://bioconductor.org/packages/release/data/annotation/html/org.Mm.eg.db.html>). Read counts were generated using Homer and normalized counts were used in DESeq2 (Bioconductor, <http://bioconductor.org/packages/release/bioc/html/DESeq2.html>) to measure differential expression. RNA-Seq raw data are available at GEO under accession number GSE143820. The candidate genes were screened from a comparison of control and stressed mice, as well as of stressed mice treated with BLS and /or scopolamine and ketamine as follows: first, the normalized counts were cut-off at 25 in at least one of the samples, then the gene lists were generated based on adjusted p-value <0.01. Lists of genes were submitted to DAVID (https://david.ncifcrf.gov) for functional enrichment analysis. For quantification of “oligodendroglia marker genes”, the top 500 genes enriched in “oligodendrocyte precursors”, “newly formed oligodendrocytes” and “myelinating oligodendrocytes” were extracted from a RNA-Seq dataset of defined cells types from the cortex (Zhang et al., 2014) using the web based interface (<https://web.stanford.edu/group/barres_lab/brain_rnaseq.html>).

**References**

Calligaro, H., Coutanson, C., Najjar, R. P. R. P., Mazzaro, N., Cooper, H. M. H. M., Haddjeri, N., et al. (2019). Rods contribute to the light-induced phase shift of the retinal clock in mammals. *PLOS Biol.* 17, e2006211. doi:10.1371/journal.pbio.2006211

Zhang, R., Lahens, N. F., Ballance, H. I., Hughes, M. E., and Hogenesch, J. B. (2014). A circadian gene expression atlas in mammals: implications for biology and medicine. *Proc. Natl. Acad. Sci.* 111, 16219–16224. doi:10.1073/pnas.1408886111
